# Supplementary material for: The Relation between Consumer Perception and Objective Understanding of Front-of-Package Nutrition Labels (FOPNLs); Results from an Online Representative Survey
Source: Nutrients. 2024 Jun 3;16(11):1751. doi: 10.3390/nu16111751 (PMC11174983; doi:10.3390/nu16111751)
Supplement: Supplementary file 1 [file nutrients-16-01751-s001.zip › nutrients-2996388-supplementary (Details for chosen food).pdf]

Details for chosen food

A. Cheese: Two different types of feta cheeses (feta cheese and reduced salt feta cheese) and a low-fat white cheese (all of which being popular among Greek adults) were chosen to assess consumer understanding of the nutrition label allocated to, in respect to the products name.

B. Yogurt & alternatives: Four different products were selected for this category based on availability and new perceived data. Specifically, the usually consumed and known as Greek yogurt with 5% fat was included as well as its low-fat alternative (2%). Two fruit- based products were also selected, one of animal origin and one of plant based to capture the objective understanding of the consumers of these low-fat of different origin, and sugar containing products, based on the plant-based trend seen today for sustainability and/or health issues [1].

C. Oils: Olive oil is frequently consumed by the Hellenic population, and is perceived as healthy, although a recent study reported that the higher the olive oil knowledge in terms of quality, the higher the probability of selecting Extra Virgin Olive Oil (EVOO) irrespective of price [2]. Two different oils, one EVOO and one containing a mixture of olive oils were therefore included for the calculation of the objective assessment score.

D. Grains: tortillas and puff pastry were selected. Two choices from the same brand were portrayed (whole wheat and refined) that differed not only in fiber content but in portion size and salt content; this was done to account for the consumers understanding and differentiation when details are provided. The puff pastry, a usually high in saturated fat food was included, as it is frequently consumed by the Hellenic population [3].

E. Fruit juices and fruit drinks: Three different products were chosen – a fruit juice 100% and two fruit drinks, one of which contained 0% added sugar. These were selected and evaluated based on the beverage guidelines developed [4] and differences observed in processing and nutrient density between these beverages [5, 6].

References

1. Craig, W.J. and U. Fresán, *International Analysis of the Nutritional Content and a Review of Health Benefits of Non-Dairy Plant-Based Beverages.* Nutrients, 2021. **13**(3): p. 842.
2. Marakis, G., et al., *Attitudes towards Olive Oil Usage, Domestic Storage, and Knowledge of Quality: A Consumers' Survey in Greece.* Nutrients, 2021. **13**(11): p. 3709.
3. Marakis, G., et al., *Changes of trans and saturated fatty acid content in savoury baked goods from 2015 to 2021 and their effect on consumers' intake using substitution models; a study conducted in Greece.* Am J Clin Nutr, 2023.

4. Popkin, B.M., et al., *A new proposed guidance system for beverage consumption in the United States.* Am J Clin Nutr, 2006. **83**(3): p. 529-42.
5. Harry Freitag Luglio Muhammad and K.M. Dickinson, *Nutrients in Beverages*, in *Nutrients, Energy Values and Health Impact of Conventional Beverages*, Alexandru Mihai Grumezescu and A.M. Holban, Editors. 2019, Academic Press. p. 41-75.
6. Pepin, A., K.L. Stanhope, and P. Imbeault, *Are Fruit Juices Healthier Than Sugar-Sweetened Beverages? A Review.* Nutrients, 2019. **11**(5): p. 1006.
