# Supplementary material for: The Relation between Consumer Perception and Objective Understanding of Front-of-Package Nutrition Labels (FOPNLs); Results from an Online Representative Survey
Source: Nutrients. 2024 Jun 3;16(11):1751. doi: 10.3390/nu16111751 (PMC11174983; doi:10.3390/nu16111751)
Supplement: Supplementary file 1 [file nutrients-16-01751-s001.zip › nutrients-2996388-supplementary.pdf]

Details for chosen food

A. Cheese: Two different types of feta cheeses (feta cheese and reduced salt feta cheese) and a low-fat white cheese (all of which being popular among Greek adults) were chosen to assess consumer understanding of the nutrition label allocated to, in respect to the products name.

B. Yogurt & alternatives: Four different products were selected for this category based on availability and new perceived data. Specifically, the usually consumed and known as Greek yogurt with 5% fat was included as well as its low-fat alternative (2%). Two fruit- based products were also selected, one of animal origin and one of plant based to capture the objective understanding of the consumers of these low-fat of different origin, and sugar containing products, based on the plant-based trend seen today for sustainability and/or health issues [1].

C. Oils: Olive oil is frequently consumed by the Hellenic population, and is perceived as healthy, although a recent study reported that the higher the olive oil knowledge in terms of quality, the higher the probability of selecting Extra Virgin Olive Oil (EVOO) irrespective of price [2]. Two different oils, one EVOO and one containing a mixture of olive oils were therefore included for the calculation of the objective assessment score.

D. Grains: tortillas and puff pastry were selected. Two choices from the same brand were portrayed (whole wheat and refined) that differed not only in fiber content but in portion size and salt content; this was done to account for the consumers understanding and differentiation when details are provided. The puff pastry, a usually high in saturated fat food was included, as it is frequently consumed by the Hellenic population [3].

E. Fruit juices and fruit drinks: Three different products were chosen – a fruit juice 100% and two fruit drinks, one of which contained 0% added sugar. These were selected and evaluated based on the beverage guidelines developed [4] and differences observed in processing and nutrient density between these beverages [5, 6].

SupplTable 1: Correct & Acceptable consumption response(s) for each food.

Food	Correct Response	Acceptable Response
Feta cheese, regular	Consume smaller portion & less frequently	
Feta cheese, reduced salt	Consume smaller portion & less frequently	
White cheese, low fat	Consume a smaller portion	Consume smaller portion & less frequently
Olive oil, extra virgin (EVOO)	Consume a smaller portion	
Olive oil, mix of refined and virgin	Consume a smaller portion	Consume smaller portion & less frequently
Yogurt with fruit, 0% fat	Consume less frequently	
Yogurt, plain 5% fat	Consume less frequently	
Plant-based alternative to yogurt with fruit	Consume less frequently	
Yogurt, plain 2% fat	May consume ad libitum	
Fruit juice refrigerated, 100%	Consume smaller portion & less frequently	
Fruit drink, refrigerated	Avoid consumption	
Fruit drink, refrigerated, 0% sugar	Consume less frequently	Avoid consumption
Tortilla wraps, whole wheat	Consume less frequently	Avoid consumption
Tortilla wraps, refined	Consume less frequently	Consume smaller portion & less frequently
Pastry dough, fresh	Avoid consumption	

SupplTable 2: FOPNL preference by Sociodemographic variables

	MTL	NUTRISCORE	NUTRINFORM	HEALTHY CHOICE	P value
**Age group**					<0.001
18-24.9	161 (55.1)	46 (15.8)	63 (21.6)	22 (7.5)	
25-59.9	429 (54.7)	97 (12.4)	169 (21.6)	89 (11.4)	
60+	67 (40.6)	25 (15.2)	33 (20.0)	40 (24.2)	
**Smoking status**					0.377
Current smoker	142 (52.4)	28 (10.3)	69 (25.5)	32 (11.8)	
Non-smoker	433 (53.6)	116 (14.4)	159 (19.7)	100 (12.4)	
Ex-smoker	92 (50.3)	25 (13.7)	43 (23.5)	23 (12.6)	
**Educational Level**					<0.001
Elementary (≤6 years)	5 (33.3)	3 (20.0)	1 (6.7)	6 (40.0)	
High school & technicians	62 (42.2)	15 (10.2)	37 (25.2)	33 (22.5)	
Graduate & Post graduate level	597 (54.5)	151 (13.8)	232 (21.2)	116 (10.6)	
**Marital Status**					<0.001
Single	349 (57.6)	83 (13.7)	129 (21.3)	45 (29.0)	
Married/cohabitting	261 (48.6)	67 (12.5)	114 (21.2)	95 (17.7)	
Divorced	33 (51.6)	7 (10.9)	14 (21.9)	10 (15.6)	
Widowed	13 (50)	4 (15.4)	4 (15.4)	5 (19.2)	
**Nutrition related degree**					<0.001
Yes	219 (60.3)	49 (13.5)	70 (19.3)	25 (6.9)	
No	447 (50.1)	119 (13.3)	201 (22.5)	126 (14.1)	
**BMI status**					
≤24.99	396 (55.2)	98 (13.7)	151 (21.0)	73 (10.2)	0.123
25.0-29.9	166 (48.4)	49 (14.3)	78 (22.7)	50 (14.6)	
≥30	99 (52.1)	21 (11.1)	39 (20.5)	31 (16.3)	
**Presence of Hypertension**	59 (48.8)	11 (9.1)	29 (23.0)	22 (18.2)	0.068
**Presence of Diabetes**	23 (46)	3 (6)	14 (28)	10 (20)	0.085
**Presence of Hypercholesterolemia**	117 (53.7)	22 (10.1)	43 (19.7)	36 (16.5)	0.064
**Presence of CVD**	21 (41.2)	6 (11.8)	14 (27.5)	10 (19.6)	0.168
**Presence of Cancer**	15 (42.9)	2 (5.7)	7 (20.0)	11 (31.4)	0.003

CVD: Cardiovascular diseas; BMI: Body Mass Index
Significance level at a=5%
P value as per between category distribution as per chi square test

	May consume ad libitum (no restrictions)			Need to consume less frequently			Consume a smaller portion			Consume smaller portion & less frequently			Avoid Consumption		
**Group**	A	B	C	A	B	C	A	B	C	A	B	C	A	B	C
Feta cheese, regular N=1328	8.4	8.6	8.9	18.1	14.3	14.0	18.8	17.1	24.0	36.1	40.7	38.2	14.5	13.8	10.9
Reduced salt feta cheese* N=1329	14.9	17.1	22.7	19.3	20.0	19.6	28.4	16.9	26.1	26.5	30.1	22.4	6.5	8.6	3.9
Low-fat white cheese* N=1327	26.8	18.7	26.7	21.7	21.1	24.1	23.1	14.7	20.6	14.0	26.6	16.0	8.0	11.2	6.8
Olive oil, extra virgin (EVOO)* N=1318	40.0	39.4	44.2	13.8	14.6	14.6	34.1	33.9	30.7	5.1	5.1	6.2	3.9	0.9	1.8
Olive oil, mix of refined and virgin* N=1317	13.8	10.8	17.4	18.2	17.0	17.4	19.9	24.3	19.0	17.5	18.1	17.2	25.2	19.5	21.2
Yogurt with fruit, 0% fat* N= 1308	40.3	54.7	49.5	21.4	15.8	14.0	7.6	6.0	8.0	7.6	6.0	9.1	19.7	10.9	15.5
Yogurt, plain 5% fat* N= 1307	24.8	37.3	24.2	25.3	22.2	26.4	23.6	16.7	25.8	15.5	12.0	12.4	7.1	7.1	8.4
Plant-based alternative to yogurt with fruit N= 1307	29.2	26.4	29.3	20.2	30.7	23.8	13.0	11.6	12.0	11.8	11.3	13.1	14.0	8.7	10.0
Yogurt, plain 2% fat N=1308	54.6	60.0	51.7	23.1	19.6	27.1	7.9	6.4	8.0	5.4	4.9	6.0	4.2	4.0	3.1
Fruit juice refrigerated, 100% N=1302*	24.3	42.9	27.8	17.6	15.4	22.4	18.3	10.9	14.0	14.6	10.9	14.4	21.5	15.2	18.9
Fruit drink, refrigerated* N=1301	11.1	8.5	12.7	19.3	31.9	24.1	12.4	13.8	13.4	18.8	14.5	17.6	33.9	26.8	29.4

Fruit drink, refrigerated, 0% sugar* N= 1301	40.1	18.3	49.0	15.6	35.3	13.6	5.9	9.8	7.8	14.9	13.2	9.8	19.3	18.1	16.3
Tortilla wraps, whole wheat N=1292	**24.9**	**48.1**	**28.5**	27.4	22.7	28.9	22.2	13.7	20.0	16.2	7.0	14.6	5.0	2.9	6.1
Tortilla wraps, refined* N=1292	12.0	11.5	11.4	25.7	42.0	26.5	18.5	15.3	22.2	23.2	15.3	22.0	15.7	10.1	15.7
Pastry dough, fresh* N= 1291	4.5	7.2	5.6	14.2	8.5	10.1	10.5	7.6	8.8	27.4	40.7	30.1	39.2	31.0	43.2

*displays between Group significant differences at a=5%, following chi square test.
Group A: Participants displayed with back of package nutritional information; Group B: Participants displayed with NutriScore information; Group C: Participants displayed with Nutrinform Battery information.
FOPNL: Front of Package Nutritional Labeling

SupplTable 3: Selected consumption response for all foods by Group (Nutrition Declaration Table or FOPNL schemes).

SupplTable 4: Proportion of population with correct response for each food by allocated Group (Nutrition Declaration Table or FOPNL schemes).

Food	Group A (%)	Group B (%)	Group C (%)	P-value[1]
Feta cheese, regular N=1328	36.1	40.2	38.8	0.437
Reduced salt feta cheese* N=1329	26.2	29.7[b]	22.3[b]	0.0244
Low-fat white cheese* N=1327	37.0	41.1	36.3	0.324
Olive oil, extra virgin (EVOO)* N=1318	34.3	33.9	30.7	0.244
Olive oil, mix of refined and virgin* N=1317	37.5	42.2	36.2	0.104
Yogurt with fruit, 0% fat* N= 1308	21.5[c]	15.9	14.0[c]	0.021
Yogurt, plain 5% fat* N= 1307	25.2	22.3	26.5	0.502
Plant-based alternative to yogurt with fruit N= 1307	20.0[a]	30.6[a,b]	23.9[b]	0.002
Yogurt, plain 2% fat N=1308	54.6	60.0[b]	51.9[b]	0.034
Fruit juice refrigerated, 100% N=1302*	18.3[a]	10.9[a]	13.8	0.017
Fruit drink, refrigerated* N=1301	33.9	26.8	29.4	0.040
Fruit drink, refrigerated, 0% sugar* N= 1301	34.9[a]	53.4[a,b]	29.8[b]	<0.001
Tortilla wraps, whole wheat N=1292	27.4	22.7	28.9	0.113
Tortilla wraps, refined* N=1292	48.9[a]	57.3[a,b]	48.4[b]	0.017
Pastry dough, fresh* N= 1291	39.2[a]	31.0[a,b]	43.2[b]	<0.001

Group significant differences at a=5%, following chi square test.
Group A: Participants displayed with back of package nutritional information; Group B: Participants displayed with NutriScore information; Group C: Participants displayed with Nutrinform Battery information.

[1]Group comparisons were derived using Anova. The model was adjusted for educational level, occupational status, sex, self-perceived nutrition knowledge and weight status. Between group differences were derived using Tukey-Kramer test following the Anova model; [b] denoting differences between Group B and Group C. No other differences were found.

FOPNL: Front of Package Nutritional Labeling

SupplFigure 1. Nutrition Labels as displayed to consumers during the survey to report whether they have observed these (each one separately) on products they have purchased in the past.

Nutrition Label	Yes	No
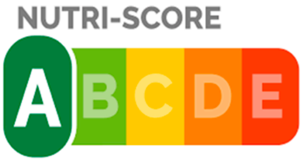		
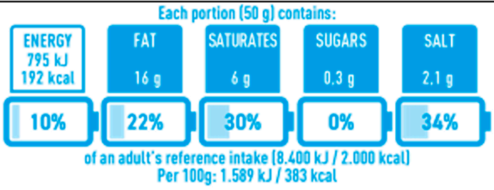		
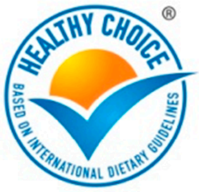		
I only read the Nutrition Declaration Tables		
I only read the ingredient list		

SupplFigure 2. FOPNL label condition of the foods displayed in the online survey by food category.

	Nutrition Information	**Feta cheese, regular**	**Feta cheese, sodium reduced**	**White cheese, low in fat**
**Group A**	Ενέργεια kcal/KJ	271 kcal/1123KJ	257kcal/1075KJ	184 kcal/767KJ
	Fat, total (g)	23 g	21 g	12 g
	Of which Fat, saturated (g)	13.8 g	15.7 g	7 g
	Carbohydrates	0 g	0 g	0 g
	Of which sugars (g)	0 g	0 g	0 g
	Fiber (g)			
	Protein (g)	0 g	0 g	0 g
	Salt (mg)	16 g	17 g	19 g
	**Nutrition Label**	2.3 g	1.5 g	1.85 g
**Group B**	NutriScore	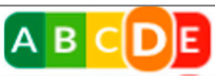	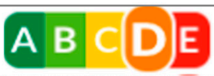	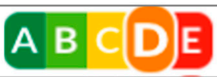
**Group C**	NutrInform Battery	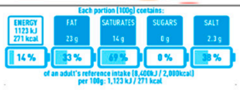	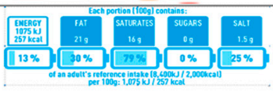	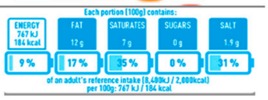

	Nutrition Information	**Olive oil, extra virgin (EVOO)**	**Olive oil, mix of refined and virgin**
**Group A**	Ενέργεια kcal/KJ	824 kcal/3416 KJ	828 kcal/3461 KJ
	Fat, total (g)	91.5 g	92 g
	Of which Fat, saturated (g)	13 g	14 g
	Carbohydrates	0 g	0 g
	Of which sugars (g)	0 g	0 g
	Fiber (g)	0 g	0 g
	Protein (g)	0 g	0 g
	Salt (mg)	0 g	0 g
**Group B**	**Nutrition Label**		
**Group C**	NutriScore	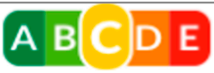	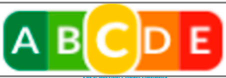
	NutrInform Battery	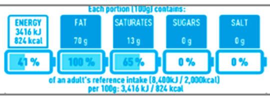	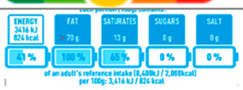

Group	Nutrition Information	Yogurt with fruit, 0% fat	Yogurt, plain 5% fat	Plant-based yogurt alternative with fruit
Group A	Ενέργεια kcal/KJ	85 kcal/354 KJ	95 kcal/398 KJ	113 kcal/474 KJ
	Fat, total (g)	0 g	5 g	5.1 g
	Of which Fat, saturated (g)	0 g	3.1 g	0.04 g
	Carbohydrates	12 g	4 g	13.6 g
	Of which sugars (g)	11 g	4 g	11.8 g
	Fiber (g)	0 g	-	2.4 g
	Protein (g)	8.8 g	8.5 g	1.6 g
	Salt (mg)	0.13g	0.18 g	0.08 g
	**Nutrition Label**			
Group B	NutriScore	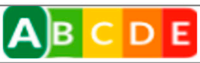	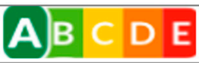	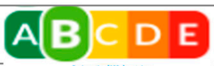
Group C	NutrInform Battery	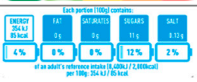	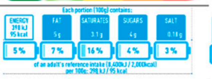	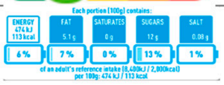

Group	Nutrition Information	Fruit juice refrigerated, 100%	Fruit drink, refrigerated	Fruit drink, refrigerated, 0% sugar
Group A	Ενέργεια kcal/KJ	44 kcal/189 KJ	49.4 kcal/205.9 KJ	1 kcal/2 KJ
	Fat, total (g)	0 g	0 g	0 g
	Of which Fat, saturated (g)	0 g	0 g	0 g
	Carbohydrates	10.7 g	11.9 g	0.1 g
	Of which sugars (g)	10.4 g	11.9 g	0.1 g
	Fiber (g)	-	0.1 g	0.1 g
	Protein (g)	0.4 g	0,2 g	0 g
	Salt (mg)	0.01 g	-	0.04 g
	**Nutrition Label**			
Group B	NutriScore	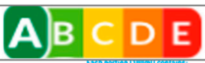	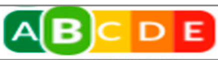	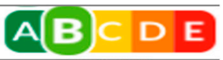
Group C	NutrInform Battery	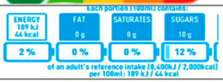	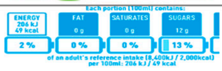	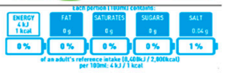

Group	Nutrition Information	Tortilla wraps, whole wheat	Tortilla wraps, refined	Pastry dough, fresh
Group A	Ενέργεια kcal/KJ	286 kcal/1207 KJ	286 kcal/1207 KJ	403 kcal/1684 KJ
	Fat, total (g)	6 g	5.2 g	27.3 g
	Of which Fat, saturated (g)	1.5 g	1,2 g	6.8 g
	Carbohydrates	46.6 g	46.6 g	33.6 g
	Of which sugars (g)	3 g	2.1 g	0.1 g
	Fiber (g)	6.4 g	1,6 g	-
	Protein (g)	8.3 g	9,1 g	5.8 g
	Salt (mg)	1 g	1 g	1.2 g
	**Nutrition Label**			
Group B	NutriScore	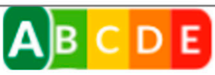	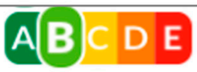	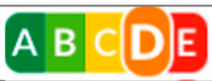
Group C	NutrInform Battery	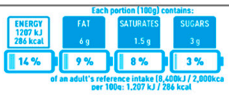	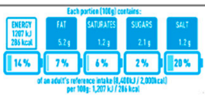	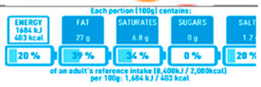

References

1. Craig, W.J. and U. Fresán, *International Analysis of the Nutritional Content and a Review of Health Benefits of Non-Dairy Plant-Based Beverages.* Nutrients, 2021. **13**(3): p. 842.
2. Marakis, G., et al., *Attitudes towards Olive Oil Usage, Domestic Storage, and Knowledge of Quality: A Consumers' Survey in Greece.* Nutrients, 2021. **13**(11): p. 3709.
3. Marakis, G., et al., *Changes of trans and saturated fatty acid content in savoury baked goods from 2015 to 2021 and their effect on consumers' intake using substitution models; a study conducted in Greece.* Am J Clin Nutr, 2023.
4. Popkin, B.M., et al., *A new proposed guidance system for beverage consumption in the United States.* Am J Clin Nutr, 2006. **83**(3): p. 529-42.
5. Harry Freitag Luglio Muhammad and K.M. Dickinson, *Nutrients in Beverages*, in *Nutrients, Energy Values and Health Impact of Conventional Beverages*, Alexandru Mihai Grumezescu and A.M. Holban, Editors. 2019, Academic Press. p. 41-75.
6. Pepin, A., K.L. Stanhope, and P. Imbeault, *Are Fruit Juices Healthier Than Sugar-Sweetened Beverages? A Review.* Nutrients, 2019. **11**(5): p. 1006.
